# The beneficial role of exercise in preventing doxorubicin-induced cardiotoxicity

**DOI:** 10.3389/fphys.2023.1133423

**Published:** 2023-03-09

**Authors:** Samantha L. Gaytan, Ahmed Lawan, Jongwha Chang, Md Nurunnabi, Sudip Bajpeyi, Jason B. Boyle, Sung Min Han, Kisuk Min

**Affiliations:** ^1^ Department of Kinesiology, College of Health Sciences, University of Texas at El Paso, El Paso, TX, United States; ^2^ Department of Biological Sciences, College of Science, University of Alabama in Huntsville, Huntsville, AL, United States; ^3^ Department of Pharmaceutical Sciences, Irma Lerma Rangel School of Pharmacy, Texas A&M University, College Station, TX, United States; ^4^ Department of Pharmaceutical Sciences, School of Pharmacy, University of Texas at El Paso, El Paso, TX, United States; ^5^ Department of Physiology and Aging, College of Medicine, Institute on Aging, University of Florida, Gainesville, FL, United States

**Keywords:** anthracycline, doxorubicin, cardiotoxicity, exercise training, oxidative stress, proteolytic systems

## Abstract

Doxorubicin is a highly effective chemotherapeutic agent widely used to treat a variety of cancers. However, the clinical application of doxorubicin is limited due to its adverse effects on several tissues. One of the most serious side effects of doxorubicin is cardiotoxicity, which results in life-threatening heart damage, leading to reduced cancer treatment success and survival rate. Doxorubicin-induced cardiotoxicity results from cellular toxicity, including increased oxidative stress, apoptosis, and activated proteolytic systems. Exercise training has emerged as a non-pharmacological intervention to prevent cardiotoxicity during and after chemotherapy. Exercise training stimulates numerous physiological adaptations in the heart that promote cardioprotective effects against doxorubicin-induced cardiotoxicity. Understanding the mechanisms responsible for exercise-induced cardioprotection is important to develop therapeutic approaches for cancer patients and survivors. In this report, we review the cardiotoxic effects of doxorubicin and discuss the current understanding of exercise-induced cardioprotection in hearts from doxorubicin-treated animals.

## Introduction

The number of cancer survivors living in the United States continues to increase annually due to the early detection of cancer and advances in treatment (Jiang et al., 2022). As of 2019, it was estimated that there are 16.9 million cancer survivors in the United States (Jiang et al., 2022). This represents approximately 5% of the population. The number is projected to reach 22.1 million by 2031 (Jiang et al., 2022). As the number of cancer survivals increases, their quality of life has become a critical issue. However, prolonged and combined cancer treatments, including cancer surgery, radiation therapy, and chemotherapy, are known to cause pain, chronic fatigue, muscle weakness, and physical dysfunction, impairing quality of life in cancer survivors (Galvao et al., 2009; Narayanan and Koshy, 2009). Approximately, 1 in 4 cancer survivors reported a decreased quality of life due to the side effects of cancer treatments (Weaver et al., 2012). Specifically, chemotherapy with anthracyclines (ANTs) has been shown to induce irreversible side effects (Tacar et al., 2013; Min et al., 2015). ANTs are a group of antineoplastic antibiotics that are highly effective chemotherapeutic agents used to treat a wide variety of cancers (Mukai et al., 2021). Unfortunately, the clinical use of ANTs is limited due to the development of cytotoxicity in the heart, resulting in cardiomyopathy and heart failure in cancer patients and survivors during cancer treatments or in even several years after cancer treatment (Kamphuis et al., 2020; Abdel-Qadir et al., 2021). Specifically, doxorubicin is among the ANTs used to manage and treat various types of malignancies and tumors (Min et al., 2015; Douedi and Carson, 2019; Lee et al., 2020; Wu et al., 2022). The primary therapeutic approach for preventing DOX-induced cardiotoxicity is to intervene with standard therapies for heart failure (Dragojevic-Simic et al., 2004; Yu et al., 2020). It has been well established that exercise training provides cardioprotective effects against DOX-induced adverse effects on the heart as a non-pharmacological cardioprotective strategy in cancer patients and survivors. This review provides an updated overview of cardiotoxicity associated with treatments of DOX and presents the current understanding of the exercise-induced protection against the cardiotoxicity.

### Anthracycline therapy

**FIGURE 1:**
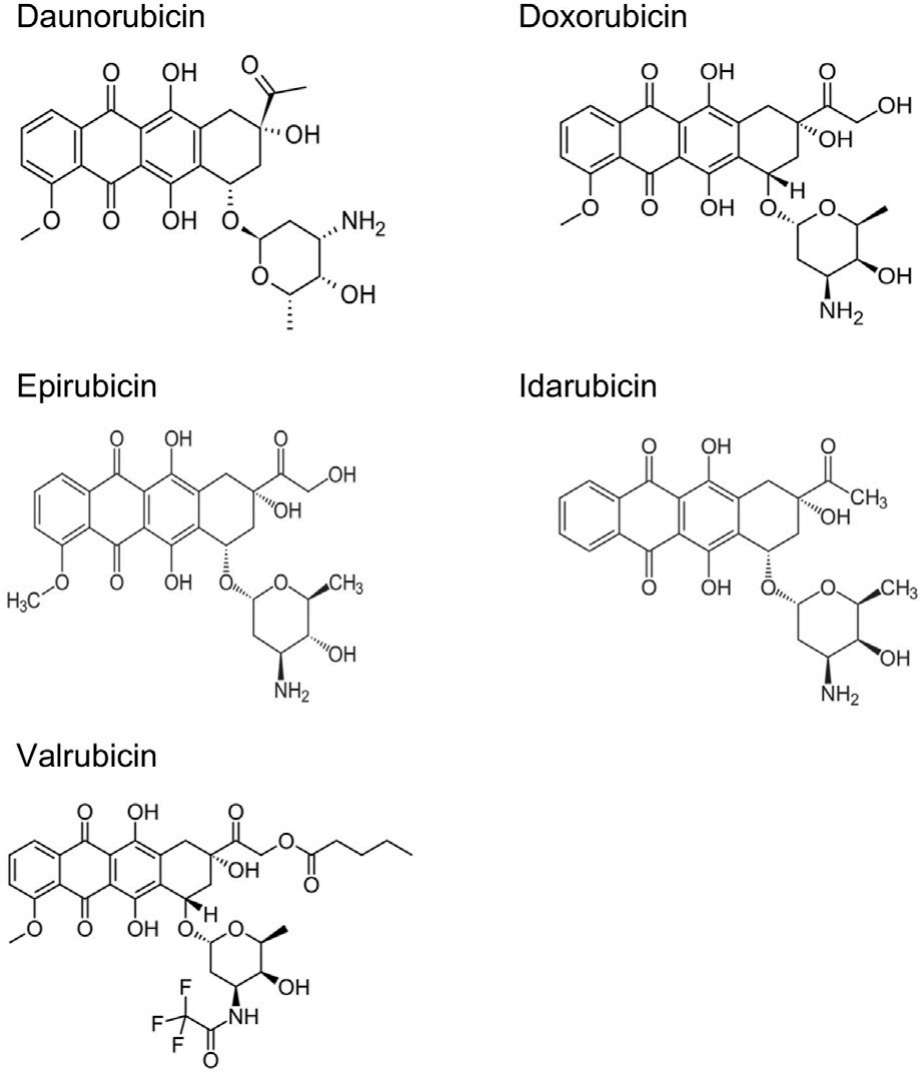
Chemical structures of main anthracyclines.

ANTs are cytostatic antibiotics that are extracted from *Streptomyces* bacterium (Fujiwara et al., 1985; Octavia et al., 2012). ANTs include daunorubicin (DAU), doxorubicin (DOX), epirubicin (EPI), idarubicin (IDA), and valrubicin (VAL) (Figure 1) (Dimarco et al., 1963; Capelôa et al., 2020). DAU was the first ANT used to treat acute pediatric leukemia in 1964 (Dimarco et al., 1963). DOX was later isolated from a mutant of *Streptomyces peucetius* in 1969 (Arcamone et al., 1969). The structural formula of DAU and DOX is very similar, except for the substitution of a hydroxyl group at the carbon 14 position in DOX (Escherich et al., 2013). DAU is utilized against acute lymphoblastic and myeloblastic leukemias, whereas DOX is more effective in lymphomas, sarcomas, and a broad spectrum of solid tumors, such as breast, lung, bladder, and bone cancers (Minotti et al., 2004; Arcamone, 2009; Tacar et al., 2013). Later on, several newer ANTs have been developed to treat multiple types of cancers (Bonfante et al., 1980; McGowan et al., 2017). Although ANTs have been successful in treating a variety of cancers, they have been associated with both acute and chronic cardiotoxicity, depending on the cumulative dose of each agent (Table 1) (Venkatesh and Kasi, 2019; Rocca et al., 2020). For example, early adverse effects of DOX have been reported to reduce the left ventricular ejection fraction within months post-treatment with a cumulative dose ≥350 mg/g^2^ (Buzdar et al., 1985). Studies with 630 breast and lung cancer patients have revealed that 32 of those 630 patients (5.1%) had DOX-induced congestive heart failure (Swain et al., 2003). Most patients with congestive heart failure were treated with a cumulative dose of ≥500 mg/m^2^. The estimated cumulative percentages of patients with DOX-induced congestive heart failure were 5%, 16% and 48% at a cumulative doses of 400 mg/g^2^, 500 mg/m^2^, and 700 mg/m^2^, respectively (Swain et al., 2003). Therefore, the dosage adjustments of ANTs are required to prevent the effects of cardiotoxicity and maximize the therapeutic effects.

**TABLE 1:** Anthracyclines, therapeutic use and maximum recommended cumulative dose.

Anthracycline	Clinical activity	Maximum cumulative dose
Daunorubicin	Treatment of acute myeloid leukemia, acute lymphoblastic leukemia, chronic myelogenous leukemia, and Kaposi’s sarcoma	550 mg/m^2^
Doxorubicin	Treatment of breast cancer, bladder cancer, stomach cancer, lung cancer, acute lymphocytic leukemia, and Kaposi’s sarcoma	550 mg/m^2^
Epirubicin	Treatment of breast cancer, stomach cancer, lung cancer, urinary tract carcinoma, and ovarian carcinoma	900 mg/m^2^
Idarubicin	Treatment of acute myeloid leukemia, acute lymphoblastic leukemia, and chronic myelogenous leukemia	150 mg/m^2^
Valrubicin	Treatment of bladder cancer	800 mg/m^2^

## Mechanisms of ANT-induced cytotoxicity

It has been recognized that ANTs act through a combination of multiple mechanisms, including 1) intercalation into DNA, 2) poisons of topoisomerase II, and 3) production of reactive oxygen species.

### DNA intercalation

**FIGURE 2:**
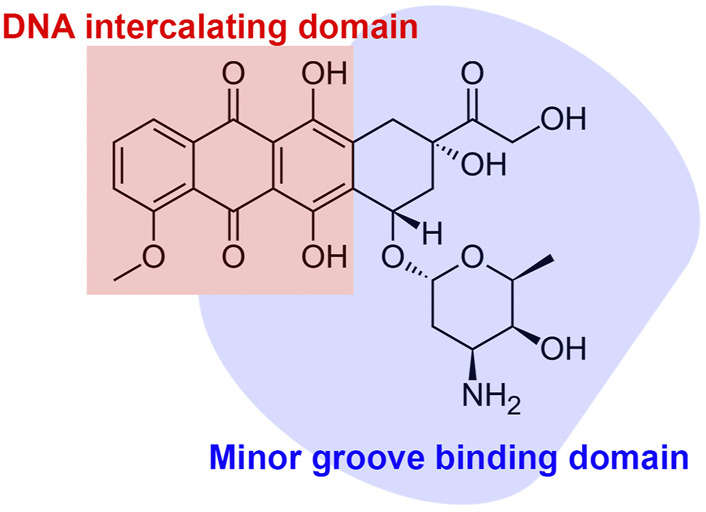
The schematic chemical structure and functional domain of anthracycline.

The activity of ANTs results in strong inhibitory effects on nucleic acid synthesis (Gewirtz, 1999; Shandilya et al., 2020). Nuclear DNA has been recognized as the primary target of ANTs (Karadurmus et al., 2021). ANTs consist of flat aromatic moieties that intercalate between DNA base pairs (Figure 2) (Frederick et al., 1990). The intercalation inhibits DNA and RNA synthesis, subsequently blocking the transcription and replication in highly replicating cells (Gewirtz, 1999). The specificity, binding affinity, and the binding mode of each ANT depend on differences in the sequence of the DNA base (Shandilya et al., 2020). The intercalation of ANTs can distort DNA and interfere with the nuclear functions in cancer cells (Wang et al., 1987; Chaires, 1990). ANTs also intercalate mitochondrial DNA (Lebrecht and Walker) to induce single or double-strand mtDNA breaks and quantitative defects in mtDNA copy number (Tewey et al., 1984; Lawrence et al., 1993; Lebrecht and Walker, 2007). Both the mutation and deletion of mtDNA-lesion compromise the synthesis of mtDNA-encoded respiratory chain subunits in the mitochondrial inner membrane, contributing to the marked mitochondrial toxicity (Lebrecht and Walker, 2007; Ashley and Poulton, 2009). Consequently, ANT-induced mitochondrial toxicity causes increased production of mitochondrial reactive oxygen species (Priya et al., 2017), which is one of the mechanisms of ANT-induced cardiotoxicity (Lebrecht and Walker, 2007).

### Topoisomerase II poisoning

Along with DNA intercalation, topoisomerase II is also considered as one of the primary targets of ANT-induced cytotoxic activity in cancer cells. Topoisomerase II is a nuclear enzyme that manages DNA tangles and supercoils by cutting both strands of the DNA helix during replication and transcription (Deweese and Osheroff, 2009). ANTs intercalated into DNA form a stable ANT-DNA-topoisomerase II ternary complex, thereby poisoning the enzyme activity. This ternary complex impends the relegation of breaks in the double-stranded DNA (Shandilya et al., 2020). As a result, ANTs induce irreversible DNA damage, leading to genomic instability and ultimately apoptotic cell death in rapidly dividing cancer cells (Li and Liu, 2001; Minotti et al., 2004; Minev, 2011). This ANT-induced topoisomerase II poisoning is also the molecular basis of cardiotoxicity. Since topoisomerase II β is present in cardiomyocytes (Capranico et al., 1992; Vejpongsa and Yeh, 2014), the inhibition of its isoform has been shown to induce long-term side effects of ANTs in cardiac muscle, resulting in cardiomyopathy (Cornarotti et al., 1996; Austin and Marsh, 1998).

### Production of reactive oxygen species

**FIGURE 3:**
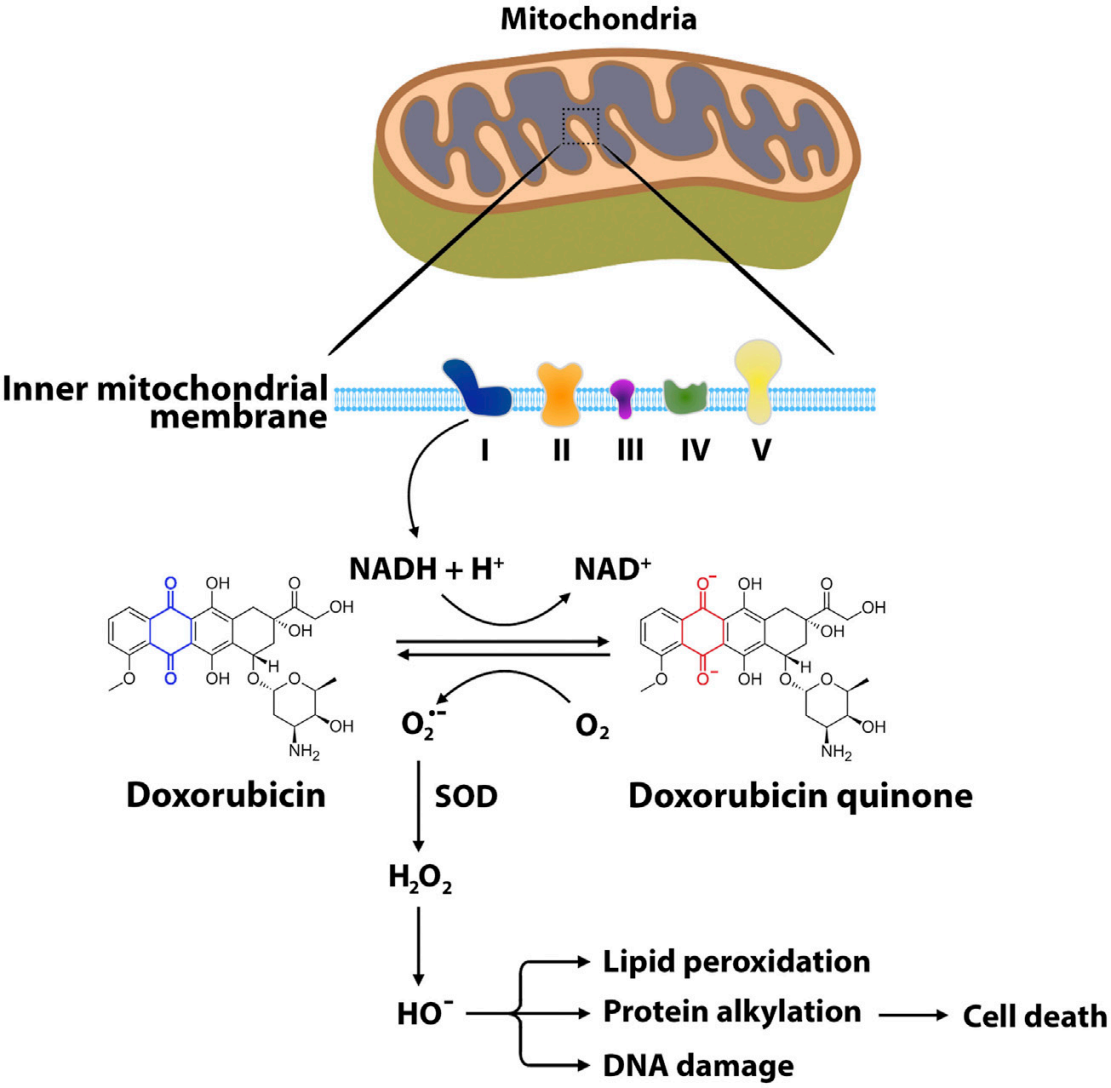
Main pathways of ANT-induced oxidative stress. The formation of reactive oxygen species begins with one-electron reduction of the quinone moiety through NADH reductase at complex I of the electron transport chain. In this reaction, the quinone ring of ANTs such as doxorubicin accepts the electron to form semiquinone, producing superoxide anion. Superoxide dismutase neutralizes the superoxide anion into hydrogen peroxide. Hydroxyl radical is produced from hydrogen peroxide through enzyme-mediated reduction-oxidation cycles. ROS interact with mitochondrial DNA, proteins, lipids, and other biomolecules, leading to cellular oxidative damage and eventually apoptosis. O_2_^•−^, superoxide radical; H_2_O_2_, hydrogen peroxide; HO^•^, hydroxyl radical; SOD, superoxide dismutase; NAD, nicotinamide adenine dinucleotide.

One of the mechanisms responsible for ANT-induced cytotoxicity is the generation of excessive reactive oxygen species (Priya et al., 2017). ROS, including superoxide radical (O_2_^•−^), hydrogen peroxide (H_2_O_2_), and hydroxyl radical (HO^•^), are byproducts of the normal metabolisms and play roles in homeostasis in normal cells (Rocca et al., 2020). However, excessively high and persistent levels of ROS result in an imbalance between the production of free radicals and antioxidant defense systems, triggering oxidative stress and cellular damage (Devasagayam et al., 2004). It has been demonstrated that mitochondria are one of the major sites for ANT-induced oxidative stress and cellular damage (Min et al., 2015). One electron is transferred from NADPH to the flavoprotein in the mitochondrial electron transport chain. The quinone ring of ANTs acts as an electron acceptor to form semiquinone, which produces superoxide anion (Berthiaume and Wallace, 2007). This reaction is catalyzed by NADH reductase at complex I in the inner mitochondrial membrane (Berthiaume and Wallace, 2007; Murabito et al., 2020). The superoxide dismutase neutralizes superoxide anion into hydrogen peroxide (Stěrba et al., 2013). The production of superoxide anion and hydrogen peroxide stimulates enzyme-mediated reduction-oxidation cycles, producing reactive and destructive hydroxyl radicals, which cause nucleic acid damage, protein alkylation, and lipid peroxidation, followed by apoptosis (Figure 3) (Simůnek et al., 2009; Angsutararux et al., 2015). In addition to mitochondrial ROS, other enzymes including NADPH oxidase (NOX) and nitric oxide synthase (NOS) also contribute to DOX-induced cardiotoxicity (Gilleron et al., 2009; Lin et al., 2019). NOX has been identified as one of the most important sources of ANT-induced ROS (Gilleron et al., 2009; Priya et al., 2017; Lin et al., 2019). NOX is a transmembrane enzyme that is located in intracellular organelles and consists of serval isoforms (Panday et al., 2015). Specifically, NOX2 has been shown to contribute to ANT-induced cardiotoxicity (Wojnowski et al., 2005; Zhao et al., 2010). Growing evidence shows that NOX2 deficiency attenuates superoxide production, preventing cardiomyocytes cell death, myocardial fibrosis, and leukocyte infiltration following DOX administration (Zhao et al., 2010; McLaughlin et al., 2017). NOS is also one of the contributing enzymes to oxidative stress and damage to cardiac muscle following DOX treatment. NOS catalyzes the conversion of L-arginine to nitric oxide (Mukai et al.) (Knowles and Moncada, 1994). Three NOS isoforms have been identified in mammals, including neuronal NOS (nNOS), cytokine-inducible NOS (iNOS) and endothelial NOS (eNOS) (Knowles and Moncada, 1994). DOX administration increases the levels of NO through the activation of eNOS and iNOS (Vásquez-Vivar et al., 1997). eNOS has been shown to catalyze NADPH-dependent superoxide formation following DOX treatment by directly binding the reductase domain of eNOS (Vásquez-Vivar et al., 1997). Eventually, the overproduction of ROS and NO generates reactive nitrogen species (RNS), which lead to cardiotoxicity following ANT treatment (Fogli et al., 2004; Štěrba et al., 2013).

## DOX-induced cardiomyopathy

DOX-induced cardiotoxicity can lead to the development of cardiomyopathy and, ultimately, congestive heart failure (Jeyaseelan et al., 1997; Singal et al., 2000). Echocardiographic analysis shows that ventricular ejection fraction, fractional shortening, and diastolic function can be reduced in the hearts treated with DOX (Lee et al., 1987; Willis et al., 2019). The dysfunction of cardiac contractility with DOX exposure results from a decrease in cardiac mass, which causes cardiac muscle atrophy and cardiac wall thinning (Min et al., 2015; Willis et al., 2019; Ye et al., 2021). The DOX-induced cardiac atrophy can be identified with an atrophic shift of myosin heavy chain isoform from alpha isoform to beta isoform and increased atrial natriuretic peptide (Willis et al., 2019). Additionally, DOX administration also causes cardiac morphological changes, such as dilated ventricles and increased myocardial fibrosis (Gyulkhasyan et al., 2019; Levick et al., 2019). DOX has also been shown to induce cardiac muscle damage through intracellular proteolytic systems (Min et al., 2015; Montalvo et al., 2020). Mammalian cells regulate the balance between protein synthesis and protein degradation, depending on the cellular demand (Rothman, 2010; Dasuri et al., 2013). Proteolytic systems stimulate protein degradation in response to cellular stresses. Although the process of protein breakdown is required for cell survival, the excessive activation of proteolytic systems in response to pathological stress can accelerate protein degradation, leading to muscular atrophy and dysfunction (Hu et al., 2008; Powers et al., 2012). Abundant evidence indicates that DOX-induced ROS production contributes to the activation of proteolytic systems. DOX-induced ROS production contributes to activation of four main proteolytic systems: 1) Ubiquitin proteasome system, 2) calpain, 3) caspase-3, and 4) autophagy.

### DOX-induced activation of ubiquitin-proteasome system

The Ubiquitin proteasome system (UPS) is an ATP-dependent proteolytic system composed of numerous ubiquitin ligase enzymes and a large proteolytic complex called the proteasome (Grune et al., 2003; Liu et al., 2016; Hyatt et al., 2019). The UPS plays a role in the protein breakdown that occurs during muscle damage (Murton et al., 2008; Powers et al., 2011). The UPS requires polyubiquitination of proteins through ubiquitin ligase enzymes, including E1 (ubiquitin-activating enzyme), E2, and E3 (Ichimura et al., 2000; Bodine et al., 2001). The polyubiquitinated proteins that are damaged or deemed unnecessary are degraded by the proteasome. Specifically, two muscle-specific E3 ligases, Muscle Atrophy F-box (MAFbx)/atrogin-1 and Muscle-Ring Finger-1 (MuRF-1), contribute to the UPS-mediated protein degradation in cardiac muscle. Numerous studies have indicated that DOX treatment stimulates UPS in cardiac muscle, leading to cardiac muscle damage (Powers et al., 2011; Derouiche et al., 2014; Min et al., 2015; Montalvo et al., 2020). Specifically, DOX treatment activates UPS through mitochondrial ROS production in cardiac muscle (Montalvo et al., 2020). Indeed, DOX administration significantly increases both mitochondrial H_2_O_2_ production and MAFbx, whereas mitochondria-targeted antioxidant protects mitochondria against DOX-induced oxidative stress and attenuates the expression of atrogin-1/MAFbx in cardiac muscle (Montalvo et al., 2020). Another study also showed that exercise preconditioning improves mitochondrial biogenesis and prevents gene expression of MuRF-1 in DOX-administrated cardiac muscle (Kavazis et al., 2014). A recent study demonstrated that a DOX dose-dependent (1–25 mg/kg) increases MuRF-1 mRNA and protein levels in myocardial tissues, accompanied by decreases in cardiac mass and cardiomyocyte cross-sectional area (Willis et al., 2019). However, mice lacking MuRF-1 were protected against DOX-induced cardiac atrophy and contractile dysfunction (Willis et al., 2019). These findings suggest that DOX administration induces pathological protein degradation through the activation of UPS in cardiac muscle.

### DOX-mediated calpain activity

Calpain is an intracellular calcium-dependent cysteine protease (Khorchid and Ikura, 2002; Vickers, 2017). Calpain exists as an inactive proenzyme in the cytosol. When intracellular calcium levels increase, the proenzyme form of calpain is converted to its active form, which cleaves cytoplasmic and nuclear substrates, leading to apoptosis (Momeni, 2011). Calpain activation has been implicated in myocardial injuries, including ischemia/reperfusion myocardial injury, pressure overload-induced cardiomyopathy, and heart failure (Li et al., 2009; Wang et al., 2018b; Wang et al., 2020). It has been demonstrated that DOX treatment causes calcium overload, which increases calpain activity (Szenczi et al., 2005; Emanuelov et al., 2010). Campos et al. established that the expression of active calpain increased in cardiomyocytes isolated from DOX-treated rats that showed dystrophin disruption in cardiac muscle (Campos et al., 2011). However, a calcium-blocking agent prevented calpain activation and preserved cardiac function. Another study also investigated the calpain-induced cardiomyopathy in rats injected with DOX (Min et al., 2015). Echocardiography analysis showed that DOX administration resulted in impaired cardiac function with decreased fractional shortening and thinning of the septal and left ventricular posterior wall. In contrast, rats treated with a calpain inhibitor prior to DOX injection attenuated cardiac dysfunction. *In vitro* experiments have also shown that DOX induces calpain activation in cardiomyocytes (Lim et al., 2004). Calpain activation in cardiomyocytes treated with DOX resulted in myofilament protein degradation and necrosis, while calpain inhibitors preserved the myofilament protein degradation (Lim et al., 2004). These studies indicate that calpain activation is one of the contributors that cause DOX-induced cardiomyopathy.

### DOX-induced activation of caspase-3

Caspases are a family of cysteine protease enzymes that contribute to programmed cell death (Shi, 2004). In healthy cells, the caspases exist as dormant pro-enzymes. The caspases undergo cleavage events in response to a death-inducing signal to release a large subunit and a small subunit that heterodimerize into the active enzyme (Roy, 2000). It has been well known that caspase-3 is a crucial mediator of apoptosis by efficiently cleaving many key cellular proteins (Porter and Jänicke, 1999). In fact, caspase-3 is highly activated during the progression of multiple forms of heart diseases (Philipp et al., 2004; Putinski et al., 2013; Hashmi and Al-Salam, 2015; Min et al., 2015). Caspase-3 activation is capable of promoting the degradation of cardiac myofibrillar proteins, such as α-actin, α-actinin, and cTnT (Communal et al., 2002). Numerous studies have established that DOX treatment induces apoptosis through the activation of caspase-3 in cardiac muscle (Michihiko et al., 2006; Pointon et al., 2010; Min et al., 2015; Sun et al., 2016) and the inhibition of caspase-3 activity attenuates DOX-induced cardiotoxicity (Wang et al., 2001; Zhang et al., 2009a; Ma et al., 2013). Although the regulation of caspase-3 activity is complex and involves several interconnected signaling pathways, both extrinsic and intrinsic pathways have been postulated to activate caspase-3 in cardiac muscle treated with DOX. DOX treatment can induce the extrinsic apoptotic pathway *via* the upregulation of death receptors (Nakamura et al., 2000). Death ligands, including FasL and TNFα, bind to their receptors, leading to caspase-8 activation. Activated caspase-8 increases caspase-3 activity, resulting in cardiomyocyte apoptosis (Zhao and Zhang, 2017; Pan et al., 2021). It is also feasible that DOX contributes to caspase-3 activation through the intrinsic apoptotic pathways by stimulating ROS production in cardiac muscle. Indeed, increased cellular levels of ROS have been reported to activate caspase-3 in a variety of cell types, including cardiomyocytes (Powers et al., 2011; Yeh et al., 2019). DOX can activate the core apoptosis regulators, such as Bax and Bak in the cytosol. The activated Bax/Bak are translocated from the cytosol to the outer membrane of mitochondria, increasing mitochondrial membrane permeability. Cytochrome c in the inner membrane of mitochondria is released into the cytoplasm (An et al., 2009). Subsequently, cytochrome c activates caspase-9, resulting in the activation of caspase-3 in DOX-treated cardiomyocytes (Wei et al., 2022). These findings many explain how DOX treatment can induce cardiomyocyte apoptosis, which causes cardiotoxicity.

### DOX-mediated autophagic signaling

Autophagy is a homeostatic process by which cytoplasmic components are degraded and recycled under normal and stress conditions through lysosomal pathways (Hansen et al., 2018). Autophagy has emerged as a major regulator of cardiac homeostasis and function. The level of autophagy in cardiac muscle is low under normal conditions, whereas it is upregulated in response to pathological stress (Nishida et al., 2009). Under physiological conditions, autophagy is essential for optimal cellular function and survival as it removes damaged or unwanted proteins and organelles. Under pathological conditions, autophagy may be stimulated to induce toxic effects (Smuder et al., 2013; Cui et al., 2021). Excessive autophagy activation can cause damage to organelles such as the mitochondria and endoplasmic reticulum, releasing compounds into the cytoplasm (e.g., cytochrome c and calcium) that can induce cell death (Nishida et al., 2008; Zhang et al., 2009b; Nishida et al., 2009). The activation of autophagy begins with the formation of a phagophore through a system of autophagy proteins (Atg proteins) (Smuder et al., 2013). The phagophore, also known as a double-membrane structure, sequesters bulk cytoplasmic components, such as abnormal intracellular proteins, excess or damaged organelles, and invading microorganisms. The phagophore expands to a sealed, double-membrane vesicle called the autophagosome (Hollenstein and Kraft, 2020). Beclin-1 plays an important role in the initial steps of autophagosome formation by mediating the localization of other Atg proteins to the phagophore (Gustafsson and Gottlieb, 2008). Elongation of the autophagosome requires the interaction of several Atg proteins (Mizushima et al., 1999; Smuder et al., 2013). Specifically, Atg4 is responsible for the cleavage of microtubule-associated protein 1A/1B-light chain 3 (LC3) to LC3-I (Yang et al., 2015). Cleaved LC3-I is conjugated by Atg7, Atg3, and Atg12-Atg5-Atg16L complex, leading to LC3-II synthesis, which is recruited to the autophagosomal membrane for the elongation (Ichimura et al., 2000). P62 is an autophagosome cargo protein that is used as a reporter of autophagy activity (Liu et al., 2016). After the formation of the autophagosome, cytoplasmic components are delivered to the lysosomes (Mizushima et al., 2002). The outer membrane of the autophagosome fuses with the lysosome to form an autolysosome (Mizushima et al., 2002). Hydrolases in lysosomes degrade the autophagosome-delivered components (Mizushima et al., 2002). Much evidence shows that the activation of autophagic signaling is associated with various forms of heart disease, including heart failure, ischemia-reperfusion injury, and metabolic cardiomyopathies (Shimomura et al., 2001; Kostin et al., 2003; Kanamori et al., 2015). This suggests that autophagy emerges as a new therapeutic target for heart disease. DOX treatment also induces autophagic signaling in cardiac muscle. Indeed, DOX-induced autophagosome and autolysosome accumulation were confirmed *in vivo* by using GFP-LC3 and mRFP-GFP-LC3 transgenic mice (Abdullah et al., 2019). In this study, both acute DOX treatment (20 mg/kg) and chronic DOX treatment (5 mg/kg every week for 4 weeks) exhibited time-dependent accumulation of LC3B II levels in cardiac muscle. Conversely, it has been reported that inhibition of autophagy *via* 3-methyladenine (3-MA) is sufficient to protect against DOX-induced autophagy, mitochondrial dysfunction, and cardiac contractile dysfunction (Lu et al., 2009; Xu et al., 2012). A proposed mechanism responsible for DOX-induced autophagy is that DOX administration results in damage to the mitochondria and induction of Beclin-1 expression, leading to accelerated autophagy and cardiomyopathy (Lu et al., 2009). Other groups also demonstrated that anti-apoptotic protein Bcl-2 can form a complex with Beclin-1 to inhibit autophagic apoptosis and autophagosome formation in cardiomyocyte (Wang et al., 2018a; Yu et al., 2022). However, DOX treatment increases the expression of Beclin-1 and decreases Bcl-2 protein expression, increasing the Beclin-1/Bcl-2 ratio, which indicates the activation of autophagic signaling and apoptosis (Smuder et al., 2013). These findings implicate that autophagy antagonists may represent a therapeutic approach for the preservation and/or maintenance of cardiac muscle function during and/or doxorubicin treatment.

## Exercise protects against DOX-induced cardiotoxicity

Since DOX treatment causes cardiac toxicity and dysfunction, pharmacological cardioprotective strategies, including chemoprotective agents (Dexrazoxane, Mesna, and Amifostine) (Dragojevic-Simic et al., 2004; Aluise et al., 2011; Yu et al., 2020) and neurohormonal therapy (β-blockers, angiotensin receptor blockers, angiotensin-converting enzyme inhibitors) have been broadly explored (Ibrahim et al., 2009; Chang et al., 2015; Beheshti et al., 2016). Alternatively, exercise has been investigated as a non-pharmacological cardioprotective strategy in cancer patients. Several studies have demonstrated that exercise training or physical activity prevents or mitigates cardiac dysfunction from DOX-induced cardiotoxicity. The cardioprotective effects of exercise may improve the chemotherapy completion rate by managing dose-limiting toxicity. Many studies have consistently shown that exercise may result in the preservation of left ventricular contractile function through various mechanisms, including increased cardiac expression of antioxidant enzymes, mitochondrial function, and reduced proapoptotic signaling (Kim and Hwang, 2015; Morton et al., 2019). The following section will summarize the potential mechanisms of exercise-induced cardioprotection against cardiotoxicity following DOX administration.

### Effect of exercise on antioxidant capacity in DOX-treated cardiac muscle

#### Antioxidant enzymes

Antioxidants are defined as substances that attenuate, delay, or prevent oxidation of another substance. Cellular antioxidants are compartmentalized in organelles and the cytoplasm to mitigate ROS and maintain redox balance (Powers and Jackson, 2008). ROS is known to decrease the activity of antioxidant enzymes, which are essential to maintain mitochondrial function by removing or neutralizing ROS produced from DOX. It has been established that exercise enhances antioxidant activities in cardiac and skeletal muscle by upregulating various cellular antioxidant systems (Ji et al., 1998; Muthusamy et al., 2012; Wang et al., 2016). Kim et al. reported that 2 weeks of aerobic exercise was sufficient to increase antioxidant enzyme activity, including superoxide dismutase (SOD) and catalase in the cardiac muscle of rats following DOX administration (Kim and Hwang, 2015). Similarly, 2 weeks of low-intensity treadmill exercise significantly increased glutathione peroxidase (GPx), attenuating left ventricular dysfunction in rats during DOX treatments. In contrast, sedentary rats treated with DOX displayed an increase in caspase-3 activity and consequently exhibited left ventricular dysfunction (Chicco et al., 2006). Exercise preconditioning also exhibited cardioprotective effects on antioxidant production in cardiac muscle. Animals subjected to 2 weeks of preconditioning prevented DOX accumulation in the mitochondria of cardiac muscle and attenuated mitochondrial ROS production, leading to the preservation of cardiac muscle contractility (Morton et al., 2019). Additionally, moderate treadmill exercise prior to DOX treatment increased the expression of the antioxidant enzymes GPx1, catalase, and manganese superoxide dismutase (Fasipe et al., 2021) in cardiomyocytes (Scott et al., 2011). Upregulation of the mentioned enzymes allows for the regulation of elevated ROS by neutralizing or removing the reactive forms and therefore, the preservation of mitochondrial function in DOX-treated cardiac muscle.

#### Non-enzymatic antioxidants

In addition to antioxidant enzymes, non-enzymatic antioxidants such as glutathione (GSH) have a critical role in the cardiac antioxidant defense system (Ascensao et al., 2007). The non-enzymatic antioxidants also interrupt and inactivate toxic free radical chain reactions. Ascensão et al. showed that DOX administration increases the amount of oxidized GSH (GSSG) in the cardiac tissue of mice, suggesting elevated oxidative byproduct release from cardiac tissue treated with DOX (Ascensão et al., 2005a). However, endurance swimming exercise reduced products of oxidative protein damage by 18.1% compared to the non-exercise group. This observation indicated that exercise induces cardiac redox adaptations that attenuate DOX-induced damage. Additionally, endurance-trained mice showed diminished levels of GSSH compared with non-trained mice. This indicates that exercise increases cardiac tissue GSH intake capacity and protects the myocardium from DOX-induced oxidative stress. Consistent with these findings, Demirel et al. found that exercise increased MnSOD and GSH concentrations, both of which remove oxidant precursors, providing antioxidant protection in cardiomyocytes (Demirel et al., 2001). In regard to mechanisms of exercise-induced non-enzymatic antioxidants, Wang et al. showed that acute exercise increased the expression of redox effector factor-1 (Ref1) and nuclear factor erythroid 2-related factor 2 (Nrf2) genes and proteins in skeletal muscle. The increased expression of these proteins was associated with mitochondrial H_2_O_2_ production and GSH and MnSOD activity (Wang et al., 2016). The authors suggest that the exercise-induced release of H_2_O_2_ stimulates the activation of the Ref1 signaling pathway. It is established that exercise-induced oxidative stress activates Nrf2, a redox-sensitive transcription factor that reduces the production of ROS by modulating the antioxidant defense systems (Muthusamy et al., 2012). Additionally, exercise increases the levels of ROS-generating NADPH oxidase-4 (Nox4) (Fasipe et al., 2021). An increase in Nox4 stimulates activation of Nrf2 which then increases the nuclear transcription of antioxidant genes, ultimately decreasing cardiomyocyte susceptibility to chemotoxic agents. These finding indicate that exercise produces adaptations in cardiac tissues that maintain the redox balance in cardiomyocytes. Specifically, exercise induces adaptations to the glutathione system and Ref1 signaling pathway, indicating the non-enzymatic antioxidants may serve as protective mechanisms against DOX toxicity.

#### Heat shock proteins

The effect of exercise-induced heat shock proteins (HSPs) on cardioprotection against DOX-induced cardiotoxicity has also been investigated. HSPs are a large family of molecular chaperones that play roles in cell survival and development by regulating protein maturation, refolding and degradation (Miller and Fort, 2018). HSPs acts as endogenous antioxidants against DOX-induced oxidative stress in the cardiac muscle. Exercise training has proven to increase the expression of HSPs, such as HSP-60, HSP-70, and HSP-72, in cardiac muscle, ameliorating the progression of DOX-induced cardiomyopathy (Ascensão et al., 2005a; Ascensão et al., 2005b; Chicco et al., 2005). Cardioprotection by HSPs may result from improved nuclear-encoded protein import into the mitochondrial matrix and protein folding ultimately reducing cellular proteolysis in cardiomyocytes (Ascensão et al., 2012). However, low-intensity exercise had no significant effect on HSPs or SOD isoforms (Chicco et al., 2006). Venkatakrishnan et al. revealed that high expression of HSP27 and its phosphorylation exhibited cardioprotective effects. This study found that phosphorylation at serine 15 and 85 of HSP 27 through p38 MAPK was a key mechanism in reduction of apoptosis in cardiac H9C2 cells treated DOX (Venkatakrishnan et al., 2006). Another study investigated the mechanisms of how HSPs prevent DOX-induced oxidative stress and cardiotoxicity (Fan et al., 2008). This study demonstrated that cardiac-specific overexpression of HSP20 attenuated acute DOX-triggered apoptosis in cardiomyocyte. This study found that HSP20 interacted with phosphorated Akt (serine 473), suggesting that the cardioprotective effect of HSP20 depends on the activity of Akt.

Together, these studies demonstrate that exercise training is an effective cardioprotective approach to prevent DOX-induced cardiotoxicity through the upregulation of antioxidant enzymes.

### Effect of exercise on mitochondrial function in DOX-treated cardiac muscle

#### DOX accumulation in mitochondria

Negatively charged cardiolipin is located on the inner membrane of the mitochondria and is essential for the activation of enzymes in the electron transport chain (ETC) (Renu et al., 2018). Doxorubicin has a cationic charge giving it a strong affinity for cardiolipin and therefore binds in an irreversible reaction (Renu et al., 2018; Smuder, 2019). The resulting cardiolipin-DOX complex allows for DOX accumulation in the mitochondria of cardiomyocytes and reduces cardiolipin availability to activate enzymes essential to complex II and IV in the ETC. Additionally, the reduced availability of cardiolipin removes a crucial binding site for cytochrome c. Consequently, oxidative phosphorylation is reduced, and the mitochondrial membrane is compromised, further enabling cardiotoxicity (Schirone et al., 2022). These reactions make mitochondria one of the major targets of DOX and therefore its dysfunction is the hallmark of DOX-induced cardiotoxicity (Wu et al., 2022). Although the mechanisms are not clear, it is possible that the protective effect of exercise is through the preservation of ETC function.

#### DOX removal from mitochondria

To preserve mitochondrial function and increase oxidative capacity, the accumulation of DOX needs to be expelled from the mitochondria. Exercise has demonstrated mitochondrial protection against DOX-induced myotoxicity. Morton et al. reported that 2 weeks of endurance exercise significantly reduced the accumulation of DOX in cardiac mitochondria and conserved mitochondrial respiratory function (Morton et al., 2019). This study investigated the effect of exercise on the expression of ATP-binding cassette (ABC) transporters (Leonessa and Clarke, 2003). These transport proteins are the regulators of chemotherapeutic drugs in cells by ATP-dependent transmembrane efflux. The authors showed that endurance exercise significantly upregulated mitochondria-specific ABC transporters located in the inner and outer mitochondrial membranes in cardiac muscle following DOX treatment. This study suggested that exercise-induced increase in the expression of ABC transport proteins may be responsible for the protective effects of exercise on the heart against DOX-induced cardiotoxicity. It is possible that ABC transporters located in the mitochondria can export the DOX accumulated in the inner membrane (Cole, 2014; Renu et al., 2018). Specifically, the transporters ABCB6, ABCB7, ABCB8, and ABCB10 are found in the inner and outer membranes of the mitochondria (Morton et al., 2019). Morton et al. showed that 2 weeks of endurance exercise preconditioning upregulated the expression and activity of all four ABC transporters. In addition to their ability to export chemotherapeutics, ABC transporters have their own unique abilities. For example, ABCB8 is known to increase mitochondrial iron export therefore reducing ROS that forms when DOX interacts with iron (Morton et al., 2019; Wallace et al., 2020). Interestingly, it has been identified that the ABC transporter, multidrug resistance protein 1 (MRP1), releases the antioxidant GSH (Cole, 2014; Renu et al., 2018). As mentioned, GSH neutralizes free radicals such as ROS and therefore, plays a critical role in maintaining oxidative capacity through the antioxidant defense system (Octavia et al., 2012). Although the mechanisms have not been elucidated, it is established that ABC transport proteins are not limited to the direct export of DOX. In fact, in addition to DOX removal, ABC transport proteins preserve mitochondrial function and reduce ROS to preserve mitochondrial function. Together, these studies suggest that one of the mechanisms by which exercise provides its protective effect may be by reducing the overall DOX present in the mitochondria.

#### Mitochondrial permeability transition

A characteristic of DOX-induced toxicity is a reduction in the mitochondrial calcium loading capacity. DOX toxicity increases calcium and phosphate overload and oxidative stress, leading to mitochondrial swelling and damage to the outer mitochondrial membrane increasing the susceptibility to permeability transition pore opening (mPTP). (Zoratti et al., 2005; Ascensão et al., 2011). When stimulated, the mPTP is responsible for the release of calcium and pro-apoptotic proteins, worsening cytotoxicity. However, exercise has been shown to defend against myocardial injury through its effect on mPTP. Ascensão et al. investigated acute endurance exercise as an intervention and showed the attenuation of calcium-induced mPTP opening in DOX-treated cardiac muscle (Ascensão et al., 2011) and chronic endurance exercise improved mitochondrial calcium tolerance (Ascensão et al., 2005b). These observations demonstrate the amelioration of mitochondrial dysfunction during and after DOX treatment. Together, these reports indicate that exercise training protects the heart from DOX-induced cardiotoxicity by protecting cardiac mitochondria-drive mPTP opening and consequently interfering with the magnitude of apoptotic pathways.

### Effect of exercise on DOX-induced proteolytic systems

#### FOXO signaling pathway

Exercise has been shown to regulate the activation of proteolytic systems. This protective effect of exercise on the attenuation of proteolytic systems is associated with the reduced Forkhead-Box O (FOXO) signaling pathway (Kavazis et al., 2014). The increased FOXO nuclear translocation causes the amplification of FOXO target genes, such atrogin-1 and MuRF-1, leading to cardiac muscle atrophy (Bodine et al., 2001; Gomes et al., 2001). DOX treatment has been shown to activate FOXO signaling through phosphorylation. Xia et al. demonstrated that DOX increases the phosphorylation of FOXO1 at Ser-249 through cyclin-dependent kinase 2 (CDK2) (Xia et al., 2020). The activated FOXO1 stimulates the transcription of proapoptotic target gene Bcl-2-interacting mediator of cell death (Bim) in cardiac muscle following DOX treatment. However, a FOXO1 inhibitor or FOXO1-specific siRNAs protected cardiomyocytes against DOX-induced apoptosis. As acute endurance exercise attenuated the activation of FOXO1 and FOXO3 in cardiac muscle following DOX administration, decreasing the activity of muscle-specific E3 ligases and ultimately apoptotic activity (Kavazis et al., 2014) could be one of the potential mechanisms involved in exercise-induced regulation of proteolytic systems, contributing to mitigating the toxicity caused by DOX treatment. It is also possible that the reduced expression of FOXO target proteins may be due to the exercise-induced upregulation of PGC-1 alpha (PGC-1α) (Sandri et al., 2006; Kavazis et al., 2014). Sandri et al. showed that overexpression of PGC-1α resulted in a reduction in atrogin-1 and MuRF-1, reducing the capacity of FOXO3 (Sandri et al., 2006). Further, downregulation of PGC-1 α has been associated with skeletal muscle atrophy (Sandri et al., 2006). Therefore, the upregulation of PGC-1 α expression may be partially responsible for the protective effect of exercise by reducing FOXO activity and inhibiting the target gene MuRF-1 (Kavazis et al., 2014).

#### Autophagic signaling

Exercise intervention also regulates autophagic signaling in hearts from DOX treated animals. Using transmission electron microscopy analysis, Wang F. et al. showed that 2 weeks of acute treadmill exercise ameliorates an increase in the number of autophagosomes and abnormal mitochondria in the heart following DOX treatment (Wang et al., 2021). Another study also revealed that exercise preconditioning inhibits DOX-induced cardiac autophagy/lysosomal system (Smuder et al., 2013). Acute preconditioning attenuated the expression of Beclin-1 and increased anti-apoptotic protein Bcl-2, thereby inhibiting autophagosome initiation. Additionally, acute preconditioning inhibited the conjugation of Atg12 to Atg5 production, which is required for the elongation of the autophagosome. Eventually, the acute preconditioning suppressed lysosomal proteases, including cathepsin B, D, and L in hearts from DOX treated animals (Smuder et al., 2013).

#### DOX-induced apoptosis

**FIGURE 4:**
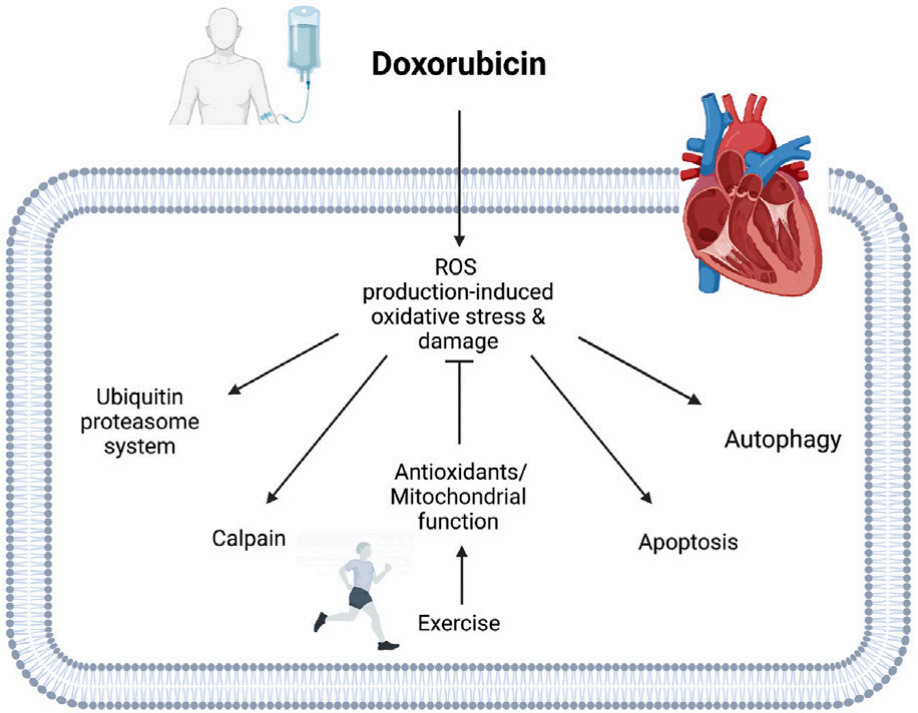
Potential mechanisms of exercise-induced cardioprotective effects against DOX cardiotoxicity. Exercise enhances cellular antioxidant defensive systems, including antioxidant enzyme, non-enzymatic antioxidant, and HSPs in hearts treated with DOX. Exercise also prevents DOX accumulation in mitochondria, improving mitochondrial function. Activated antioxidant systems and enhanced mitochondrial function mitigate DOX-induced oxidative stress and damage in cardiac muscle. The protective mechanisms of exercise can protect cardiac muscle by attenuating DOX-induced proteolytic systems.

Exercise training also prevents DOX-induced apoptosis in the heart. Alihemmati et al. exercised male Wistar rats with alternating intervals of high and low training for 1 h a day, 5 days a week, for 6 weeks using a rodent treadmill. The interval training cycle lasted 7 min with high intensity exercise (85–90% VO_2max_) for 4 min and the low intensity exercise (65–75% VO_2max_) for 3 min. After the 6 weeks of interval exercise training, the rats received 20 mg/kg of DOX (Alihemmati et al., 2019). This study showed that the 6 weeks of interval training reduces Bax protein expression and increases Bcl-2 protein expression, leading to a decreased Bax/Bcl-2 ratio in hearts from DOX treated animals, thereby reducing apoptosis. The authors also confirmed the effect of interval training on cardiomyocyte apoptosis through TUNEL staining. The 6 weeks of interval training reduced TUNEL-positive apoptotic cells in hearts from DOX treated animals. The authors also examined the activity of microRNAs that modulate the damage pathways in cardiomyocytes in response to heart disease. The interval training attenuated the overexpression of microRNA-499 in hearts from DOX treated animals, which is a potential biomarker for apoptotic effects in cardiomyocytes. Exercise has been shown to inhibit both intrinsic and extrinsic apoptotic pathways in DOX-treated animals. In this regard, Magalhães et al. reported that exercise preconditioning prevents the expression of caspase-9 and caspase-3 proteins in animals treated with DOX, indicating that exercise attenuates the DOX-induced intrinsic apoptotic pathway (Magalhães et al., 2017). Another study revealed that treating animals with DOX significantly increased the activity of both caspase-8 and caspase-9 in cardiac muscle, whereas 12 weeks of endurance treadmill training prevented the increases in cardiac muscle following DOX treatment, resulting in decreased caspase-3 activity (Marques-Aleixo et al., 2018). Collectively, these studies suggest that exercise training can prevent cardiac muscle degradation by alleviating the DOX-induced proteolytic systems (Figure 4).

## Conclusion

Cancer therapy has significantly improved, and as a result, the lifespan of cancer survivors has increased. Therefore, it is important to develop countermeasures to prevent chemotherapy-induced cardiotoxicity that impairs the quality of life for survivors. Various mechanisms are involved in DOX-induced cardiotoxicity. Given the abundance of reports indicating that exercise can result in a protective phenotype of the heart against the cardiotoxicity, exercise therapy as a non-pharmacological intervention can be an effective clinical approach to prevent or reverse the side effects of chemotherapy.

Investigations into the mechanisms responsible for exercise-induced cardioprotection against the cardiotoxicity from chemotherapy are still in the early stages. Thus, further research is required to provide comprehensive evidence considering various exercise type, intensity, and duration to develop exercise training protocols for cancer patients and survivors.
